# Dual-targeting CD73/PD-L1 bifunctional inhibitor: a promising cancer immunotherapy strategy

**DOI:** 10.3389/fimmu.2026.1898645

**Published:** 2026-07-23

**Authors:** Jing-Jing Du, Sen Wu, Shiyun Cheng, Xiaobo Zeng, Binbin Cheng

**Affiliations:** 1 Hubei Provincial Key Laboratory of Occurrence and Intervention of Kidney Diseases, Hubei Provincial Engineering Research Center of Immunotherapy Drugs for Renal Tumors, Hubei Polytechnic University, School of Medicine, Huangshi, Hubei, China; 2 Hubei Institute for Drug Control, Hubei Institute of Biological Products, Wuhan, Hubei, China

**Keywords:** bifunctional inhibitors, cancer, CD73, immunotherapy, PD−1, PD−L1

## Abstract

**Objectives:**

This work aims to design and characterize a novel bifunctional small molecule that simultaneously targets PD-L1 and CD73 to enhance the therapeutic efficacy of tumor immunotherapy.

**Methods:**

Multiple methodologies were integrated for compound screening and biological characterization, including computer-aided molecular docking, homogeneous time-resolved fluorescence (HTRF) binding assay, surface plasmon resonance (SPR), and PD-1/PD-L1 NFAT reporter cell assay.

**Results:**

The lead compound CP-1 exhibited potent dual-target inhibitory activities. It blocked the PD-1/PD-L1 interaction with an IC_50_ of 10.27 nM and suppressed CD73 activity with an IC_50_ of 300.2 nM. Molecular docking simulations revealed that CP-1 stably binds to the functional domains of PD-L1 and CD73 via specific non-covalent interactions. Cellular functional assays further demonstrated that CP-1 effectively restored T cell function in the PD-1/PD-L1 reporter system, with an EC_50_ of 0.9 μM.

**Conclusions:**

CP-1 exhibits balanced, dual-nanomolar inhibitory activity against PD-L1 and CD73 and displays potent immunomodulatory effects at the cellular level. It serves as a promising lead candidate for developing novel bifunctional agents to advance tumor immunotherapy.

## 1 Introduction

Immune checkpoint blockade targeting the PD-1/PD-L1 axis restores antitumor immunity by disrupting binding between PD-1 on effector T cells and its ligand PD-L1 on tumor cells, thereby reversing T cell exhaustion and restoring cytotoxic lymphocyte function (1, 2). Nevertheless, the clinical efficacy of PD-1/PD-L1 inhibitors remains unsatisfactory due to intrinsic and acquired therapeutic resistance, with objective response rates generally limited to approximately 20% (3–5). Such suboptimal therapeutic outcomes indicate that PD-1/PD-L1 monotherapy alone cannot fully reverse the multifaceted immunosuppressive tumor microenvironment (TME) (6, 7). PD-L1 is broadly expressed not only on tumor cells but also on antigen-presenting cells and stromal cells within the TME. Upon binding to PD-1, PD-L1 transmits strong inhibitory signals to repress T cell activation, proliferation, cytokine secretion, and cytolytic capacity, ultimately facilitating tumor immune evasion (8–10). In addition, PD-L1 impairs dendritic cell maturation and antigen presentation, further dampening the initiation and amplification of adaptive antitumor immune responses (11, 12). As systematically summarized in a recent pan-cancer clinical analysis, the narrow therapeutic window and frequent primary/secondary resistance severely restrict the clinical benefits of single-agent PD-L1 blockade, even across multiple solid tumor types. To address these unmet clinical needs, additional immunosuppressive pathways that coexist within the TME should be co-targeted with PD-L1 to restore robust antitumor immune responses (13).

**Figure 1:**
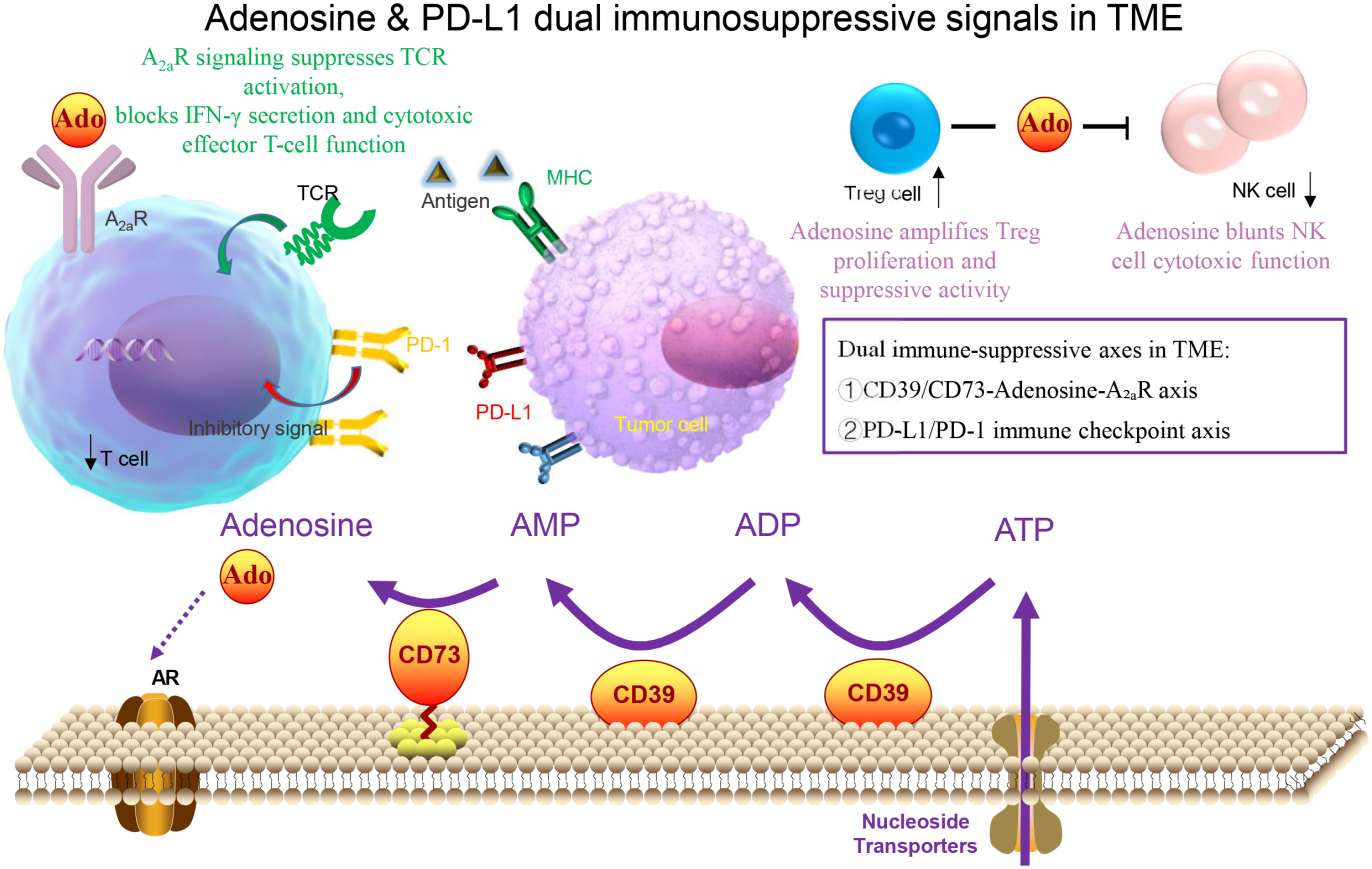
Schematic diagram of two synergistic immunosuppressive axes within the tumor microenvironment. Extracellular ATP is sequentially hydrolyzed by membrane-bound CD39 and CD73 to produce adenosine, which transmits inhibitory signals to T cells via A_2a_ receptors. Separately, tumor-expressed PD-L1 binds PD-1 on T lymphocytes, thereby triggering T cell exhaustion. The two non-redundant inhibitory signaling pathways are illustrated in the diagram (24).A_2a_R: A_2a_ adenosine receptor; MHC: major histocompatibility complex; AR: adenosine receptor.

Among the dominant resistance-driving axes independent of PD-L1 signaling, the extracellular adenosine metabolic network has been validated as a core suppressor of tumor immunity via multiple single-cell and *in vivo* mechanistic studies. Extracellular adenosine accumulates in hypoxic tumor lesions and acts as a broad-spectrum immune suppressor, dampening nearly all effector immune subsets and creating a complementary immunosuppressive layer distinct from PD-L1-mediated checkpoint inhibition (14, 15). CD73, a rate-limiting ectoenzyme in the extracellular adenosine metabolic cascade, has been identified as a central immunosuppressive mediator and a major driver of resistance to anti-PD-1/PD-L1 therapies. CD73 generates abundant extracellular adenosine, which independently suppresses CD8^+^ T cell cytotoxicity, expands Treg/MDSC suppressor populations, and upregulates alternative T cell exhaustion markers—all pathways unaffected by PD-L1 blockade alone. Clinical and preclinical data confirm that elevated tumoral CD73 predicts poor anti-PD-L1 responses, and that CD73 inhibition robustly restores immunotherapy efficacy, confirming its dominant role in treatment failure (5, 16). Together with CD39, CD73 catalyzes the sequential hydrolysis of extracellular ATP to AMP, which is then degraded to the immunosuppressive adenosine (Ado) (17, 18). CD73 generates high levels of tumor-derived adenosine (ADO) to drive anti-PD-L1 resistance by activating all four P1 adenosine receptors (A_1_R, A_2_AR, A_2_BR, A_3_R) on immune and tumor cells. ADO primarily signals through T cell A_2_AR to directly inhibit T cell activation and effector function; Gs-coupled A_2_A/A_2_B receptors boost cAMP to suppress effector T cells and expand immune suppressors, whereas Gi-associated A_1_/A_3_ receptors further amplify T cell exhaustion and tumor progression. These redundant adenosine signaling axes persist despite PD-L1 blockade and jointly dampen the efficacy of immunotherapy. ADO also weakens antigen-presenting cell activity, amplifies the suppressive capacity of regulatory T cells (Tregs), and attenuates the cytotoxicity of natural killer (NK) cells, collectively forming a layered, self-amplifying immunosuppressive niche (19, 20). Notably, the CD73–adenosine axis and PD-1/PD-L1 pathway exert nonredundant yet synergistic immunosuppressive effects, and their reciprocal regulatory crosstalk within the TME has been thoroughly characterized by mechanistic pathological investigations (21). Briefly, PD-L1 upregulation further accelerates CD73 expression on both tumor and stromal cells, while adenosine signaling, in turn, stabilizes surface PD-L1 protein, amplifying immune escape signals and forming a mutually reinforcing immunosuppressive circuit. This reciprocal regulatory relationship provides a clear mechanistic rationale explaining why simultaneous dual blockade of PD-L1 and CD73 would produce superior antitumor efficacy compared with individual monotherapy regimens. Their co-expression within the TME is frequently observed and strongly correlates with advanced tumor staging and poor clinical prognosis. Accumulating preclinical and emerging clinical evidence confirms that selective CD73 inhibition significantly improves the therapeutic response to PD-L1 blockade (**Figure 1**) (22, 23). Collectively, these mechanistic findings provide a solid theoretical basis for co-targeting CD73 and PD-L1 to overcome immunotherapy resistance and achieve durable antitumor immunity.

**Figure 2:**
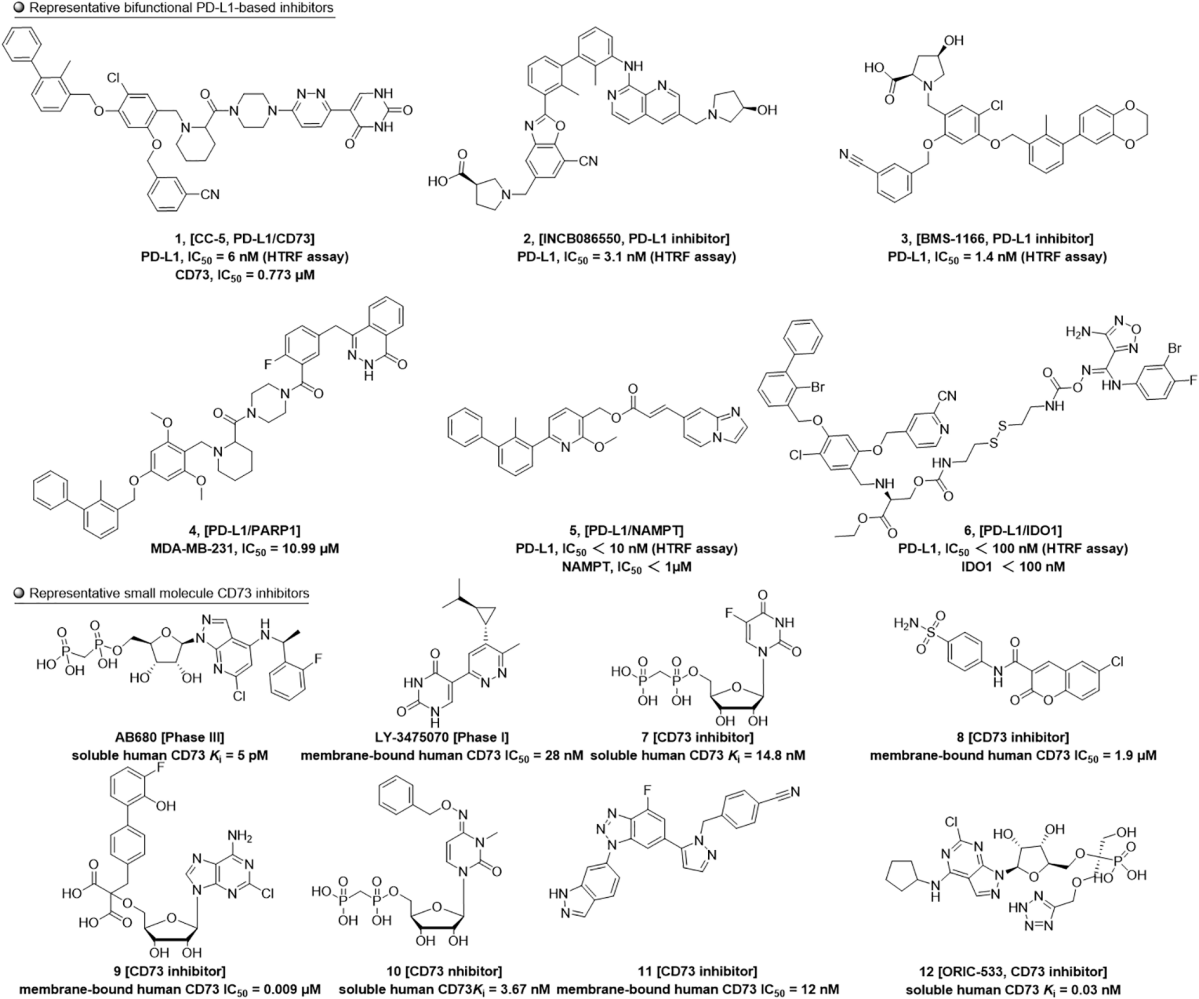
Summary of reported bifunctional small-molecule immunomodulators targeting PD-L1 or CD73. Multiple single-molecule agents co-targeting PD-L1 alongside epigenetic, metabolic, or immune-related proteins have demonstrated robust preclinical antitumor efficacy, while a panel of selective CD73 small-molecule inhibitors is currently under preclinical or clinical assessment. These results demonstrate the therapeutic potential of dual immunomodulatory approaches against malignancies.

Building on this mechanistic rationale, previous studies have validated the therapeutic potential of PD-L1-centered dual-targeting strategies (10, 25, 26). For instance, bifunctional inhibitors co-targeting PD-L1/CXCL12 or PD-L1/tubulin elicit stronger antitumor activity relative to PD-L1 monotherapy. Moreover, a growing panel of rationally designed bifunctional small molecules—including PD-L1/HDAC, PD-L1/NAMPT, PD-L1/PARP1, and PD-L1/IDO1—have exhibited robust preclinical activity, jointly demonstrating the feasibility, versatility, and therapeutic potential of PD-L1-based dual-immunomodulatory agents (**Figure 2**) (27–29). In parallel, CD73-targeted therapeutic modalities have undergone rapid development: multiple small-molecule and biological CD73 inhibitors are currently under preclinical or clinical evaluation (**Figure 2**). Among these, the CD73 inhibitor LY-3475070 is being assessed in a Phase I clinical trial in combination with pembrolizumab (30, 31). Importantly, preclinical data reveal that PD-L1/CD73 bispecific antibodies generate superior synergistic antitumor immunity compared with single-agent treatment or conventional antibody combination regimens (32). While antibody dual immunotherapies exhibit superior target specificity and potency compared with small molecules, their clinical translation is limited by inherent limitations, including immunogenicity, poor penetration into tumor tissue, and unfavorable pharmacokinetic profiles. In contrast, small-molecule dual inhibitors possess unique advantages: enhanced tissue permeability, oral bioavailability, scalable chemical synthesis, and favorable pharmacokinetic–pharmacodynamic properties. These characteristics collectively improve druggability and translational prospects (12, 18, 33). Given the well-documented functional synergy between PD-L1 and CD73 in shaping an immunosuppressive TME, herein we report the rational design, chemical synthesis, and systematic biological characterization of a novel bifunctional small-molecule inhibitor that simultaneously engages PD-L1 and CD73.

## 2 Materials and methods

### 2.1 Chemical general methods

All reagents and solvents were purchased from commercial suppliers and used without further purification unless otherwise specified. INCB086550 (*InvivoChem*, Catalog No.: V4418) was utilized as a positive control. Reaction progress was monitored by thin-layer chromatography (TLC), with visualization under UV light (254 nm) and/or iodine vapor staining. Column chromatography was performed on silica gel (300–400 mesh, Qingdao Haiyang Chemical Co., Ltd.) using gradient elution. ¹H and ¹³C NMR spectra were acquired on a Bruker AV-400 spectrometer (400 MHz for ¹H NMR; 101 MHz for ¹³C NMR), with chemical shifts reported in parts per million (ppm) relative to tetramethylsilane (TMS) as internal standard. High-resolution mass spectra (HRMS) were recorded on a Waters QTOF mass spectrometer in electrospray ionization (ESI) positive mode. HPLC analysis was performed on a Shimadzu LC-20AD/T system equipped with a DGU-20A5R online degasser, a CT0-20A column oven and an SPD-20A UV-Vis detector. Chromatographic separation was achieved using an XBridge C18 column (50 mm × 4.6 mm, 5.0 μm). Mobile phase A was deionized water, and mobile phase B was acetonitrile containing 0.1% trifluoroacetic acid (TFA). Gradient elution was carried out at a flow rate of 0.65 mL/min with the following elution program: 0–1 min, maintained at 30% mobile phase B; 1–10 min, linear increase of mobile phase B to 60%; 10–17 min, held at 60% mobile phase B; 17–25 min, linear decrease of mobile phase B back to 30%; 25–30 min, kept constant at 30% mobile phase B. The column temperature was set to 30°C, the injection volume to 5 μL, and the detection wavelength to 254 nm.

### 2.2 Characterization and analytical test results of the target compound

Compound CP-1 ^1^H NMR (400 MHz, *d*6-DMSO) δ 11.71 (s, 2H), 11.62 (s, 2H), 10.61 (s, 2H), 8.63 (d, *J* = 9.0 Hz, 2H), 8.57 (d, *J* = 4.7 Hz, 2H), 8.32 (d, *J* = 9.0 Hz, 2H), 7.78 (d, *J* = 7.9 Hz, 2H), 7.35 (t, *J* = 7.8 Hz, 2H), 7.05 (d, *J* = 7.4 Hz, 2H), 2.05 (s, 6H). ^13^C NMR (101 MHz, *d*6-DMSO) δ 163.27, 161.55, 156.89, 151.33, 151.05, 144.53, 142.30, 136.44, 130.26, 127.08, 126.91, 126.27, 126.14, 124.17, 107.51, 15.13. HRMS m/z: calcd for C_32_H_25_N_10_O_6_ [M+H]^+^ 645.1959, found 645.1960. HPLC: *t*_R_ 13.71 min, purity 98.51%.

### 2.3 *In vitro* PD-1/PD-L1 binding HTRF assay

The inhibitory activity of test compounds against the PD-1/PD-L1 protein–protein interaction was quantitatively evaluated using a homogeneous time-resolved fluorescence (HTRF) binding kit (Catalog No.: 64PD1PEG, PerkinElmer) following the manufacturer’s standard protocol. In this assay, recombinant Tag1-PD-L1 and Tag2-PD-1 proteins form a binary complex that generates a Förster resonance energy transfer (FRET) signal between the HTRF donor (anti-Tag1-Europium cryptate) and the acceptor (anti-Tag2-XL665). FRET intensity positively correlates with the extent of PD-1/PD-L1 complex formation. Three experimental groups were set up: a vehicle negative control, a known PD-1/PD-L1 inhibitor, BMS-202, as the positive control, and serial dilutions of test compounds, with three technical replicates per group. Briefly, 2 μL of diluent buffer (negative control) or serially diluted compound solution was added to each well of a low-volume 384-well plate, followed by sequential addition of 4 μL diluted Tag1-PD-L1 and 4 μL diluted Tag2-PD-1. Next, 10 µL of the pre-mixed anti-Tag1-Eu3+ and anti-Tag2-XL665 was added, the plate was sealed, and the plate was incubated for 2 hours at room temperature. Fluorescence intensities at 665 nm (acceptor emission) and 620 nm (donor emission) were read on an HTRF^®^ compatible reader.

### 2.4 *In vitro* CD73 binding assay

The 5× CD73 assay buffer (R&D Systems; 25 mM Tris, pH 7.4, 5 mM MgCl_2_) and AMP substrate were thawed on ice before use. For each well of a low-volume 384-well plate: 2.5 μL of test compound or vehicle control, 10 μL CD73 enzyme solution (0.5 ng total protein), and 7.5 μL reaction master mix (3 μL 5× CD73 buffer + 4.5 μL ultrapure water) were added sequentially. The mixture was preincubated at 37°C for 60 min, after which 5 μL AMP substrate (500 μM) was supplemented to initiate the enzymatic reaction. After incubation at 37°C for 20 min, 50 μL colorimetric detection reagent was added to each well. Plates were briefly shaken and incubated at 37°C for 15 min, and absorbance values at 630 nm were finally recorded.

### 2.5 PD-1/PD-L1 NFAT reporter bioassay

PD-1/NFAT/Jurkat reporter cells and CHO/PD-L1/TCR cells were purchased from Conradbio Co., Ltd. and cocultured to assess the cellular functional activity of PD-L1 inhibitors. CHO/PD-L1/TCR cells stably overexpress TCR ligand and cell-surface PD-L1, while modified Jurkat T cells overexpress PD-1 alongside an NFAT promoter-driven luciferase reporter. Both cell lines were maintained in complete RPMI 1640 medium. CHO/PD-L1/TCR cells were seeded into 96-well plates at a density of 3.5 × 10^4^ cells/mL and cultured at 37°C with 5% CO_2_ for 24 h before treatment. On the day of the assay, culture supernatants were discarded, and serial dilutions of test compounds were overlaid onto the cell monolayers. Plates were incubated at 37°C with 5% CO_2_ for 0.5 h, then 4.0 × 10^4^ PD-1/NFAT/Jurkat reporter cells were added per well. After 5 h of coculture at 37°C with 5% CO_2_, plates were equilibrated to room temperature for 10 min, and 100 μL Bio-Glo luciferase substrate was added to each well. EC_50_ and maximum relative luminescence (RLUmax) values were determined by fitting raw data to the Hill equation.

### 2.6 Surface plasmon resonance (SPR) assay

Human and murine PD-L1 proteins were purchased from Sino Biological: human PD-L1 (Catalog No. 10084-HNAH), murine PD-L1 (Catalog No. 50010-M08H). Human and murine CD73 proteins were obtained from Novoprotein: human CD73 (Catalog No. CW84), murine CD73 (Catalog No. CI98). SPR measurements were performed on a PlexArray HT SPRi system. Briefly, human/murine PD-L1 protein was diluted to 0.5 mg/mL in sterile water and covalently immobilized onto bare gold PlexArray nano-capture sensor chips via amide coupling. Serial concentrations of CP-1 were injected into the flow cell (injection volume = 30 μL, flow rate = 30 μL/min) using a nonpulsatile piston pump to record binding responses. The processed curves were globally fitted to the 1:1 Langmuir binding model. Kinetic data collection and fitting were performed using PlexArray Analyzer software.

### 2.7 *In ex vivo* MDA-MB-231–CD3/CD28 T cell co-culture systems

The *ex vivo* T cell-mediated antitumor activity of CP-1 was assessed via the Cell Counting Kit-8 (CCK-8) cell viability assay. Commercially available human peripheral blood mononuclear cells (PBMCs) were purchased from KEFAN Culture Collection. For T cell activation, purified CD3^+^ T cells were cultured in RPMI-1640 complete medium supplemented with 10% fetal bovine serum, 1% penicillin–streptomycin, 50 μM 2-mercaptoethanol, and 100 U/mL recombinant human IL-2, and stimulated with anti-CD3/CD28 magnetic co-activation beads at a bead-to-cell ratio of 1:1, consistent with the established T cell activation workflow. T cells were incubated for 72 h at 37°C in 5% humidified CO_2_ to achieve full activation; freshly activated T cells without additional long-term expansion were then used in subsequent co-culture assays. MDA-MB-231 tumor cells (1 × 10^5^ cells per well) were seeded in 96-well plates and incubated overnight in DMEM complete medium (10% FBS, 1% penicillin–streptomycin) to allow cell adherence. The next day, pre-activated CD3/CD28-activated T cells (5 × 10^5^ cells per well, tumor: T cell ratio = 1:5) were added to generate tumor–T cell co-cultures, followed by treatment with serially diluted CP-1 at concentrations ranging from 0.1 μM to 1 μM. The co-culture system was maintained in a mixed RPMI-1640/DMEM complete medium at 37°C in a 5% CO_2_-humidified incubator. After 24 h of coincubation, CCK-8 reagent was added to each well and incubated for 2 h; absorbance at 450 nm was measured to calculate relative cell viability for all groups, following standard CCK-8 detection guidelines.

### 2.8 Cellular CD73 assay

Cell-surface CD73 enzymatic activity was quantified by LC-MS/MS detection of adenosine generated from the AMP substrate. MDA-MB-231 cells were cultured in complete DMEM, seeded at 10,000 cells per well in 96-well plates, and incubated overnight to promote adherence. Serially diluted CP-1 was prepared in DMSO and further diluted into DMEM containing 10% human plasma to prepare 10× working stock solutions. Cell culture medium was discarded, and cells were incubated with compound dilutions for 15 min at 37°C, 5% CO_2_, followed by the addition of AMP and EHNA to final concentrations of 10 μM and 5 μM, respectively. After 1 h, cell supernatants were harvested and mixed with methanol–acetonitrile (1:1, v/v, 0.1% formic acid). Adenosine and AMP concentrations were measured by LC-MS/MS.

### 2.9 Molecular docking simulations

Molecular docking calculations were executed using AutoDock Vina. High-resolution crystal structures of human PD-L1 (PDB ID: 6RPG) and human CD73 (PDB ID: 6XUE) were retrieved from the RCSB Protein Data Bank. For receptor preparation, water molecules, redundant heteroatoms, and extra protein chains were removed using AutoDock Tools. Missing hydrogen atoms were supplemented, and Gasteiger partial atomic charges were assigned before exporting processed receptors as PDBQT files. The ligand CP-1 was *de novo*-constructed and subjected to geometry optimization to obtain a minimized 3D conformation, which was then imported into AutoDock Tools. Polar hydrogens were added, Gasteiger charges were calculated, and all rotatable bonds were defined before exporting the ligand as a PDBQT file. Binding pockets were defined based on structural and functional evidence: the grid box for PD-L1 was centered on the homodimer interface cavity (X = -0.73, Y = -25.22, Z = -15.54, 20 × 20 × 20 Å), whereas the grid box for CD73 covered the catalytic zinc-binding active site (X = 26.44, Y = 77.31, Z = 29.51, 20 × 20 × 20 Å). Cubic grid boxes were generated for each target, along with the corresponding grid and docking parameter files. Twenty independent docking runs were performed for each protein–ligand complex, and the lowest-energy binding pose was selected as the representative binding conformation for analysis.

### 2.10 Statistical analysis

All quantitative data were obtained from three independent biological replicates. Prior to one-way analysis of variance (ANOVA), the normality of the data distribution and homogeneity of variances were assessed to satisfy the prerequisite assumptions. Following significant overall ANOVA results, Tukey’s *post-hoc* multiple comparison test was conducted for pairwise intergroup comparisons. Significance thresholds were defined as *P < 0.05, **P < 0.01, ***P < 0.001, ****P < 0.0001.

## 3 Results

### 3.1 Rational design of dual PD-L1/CD73 inhibitors

**Figure 3:**
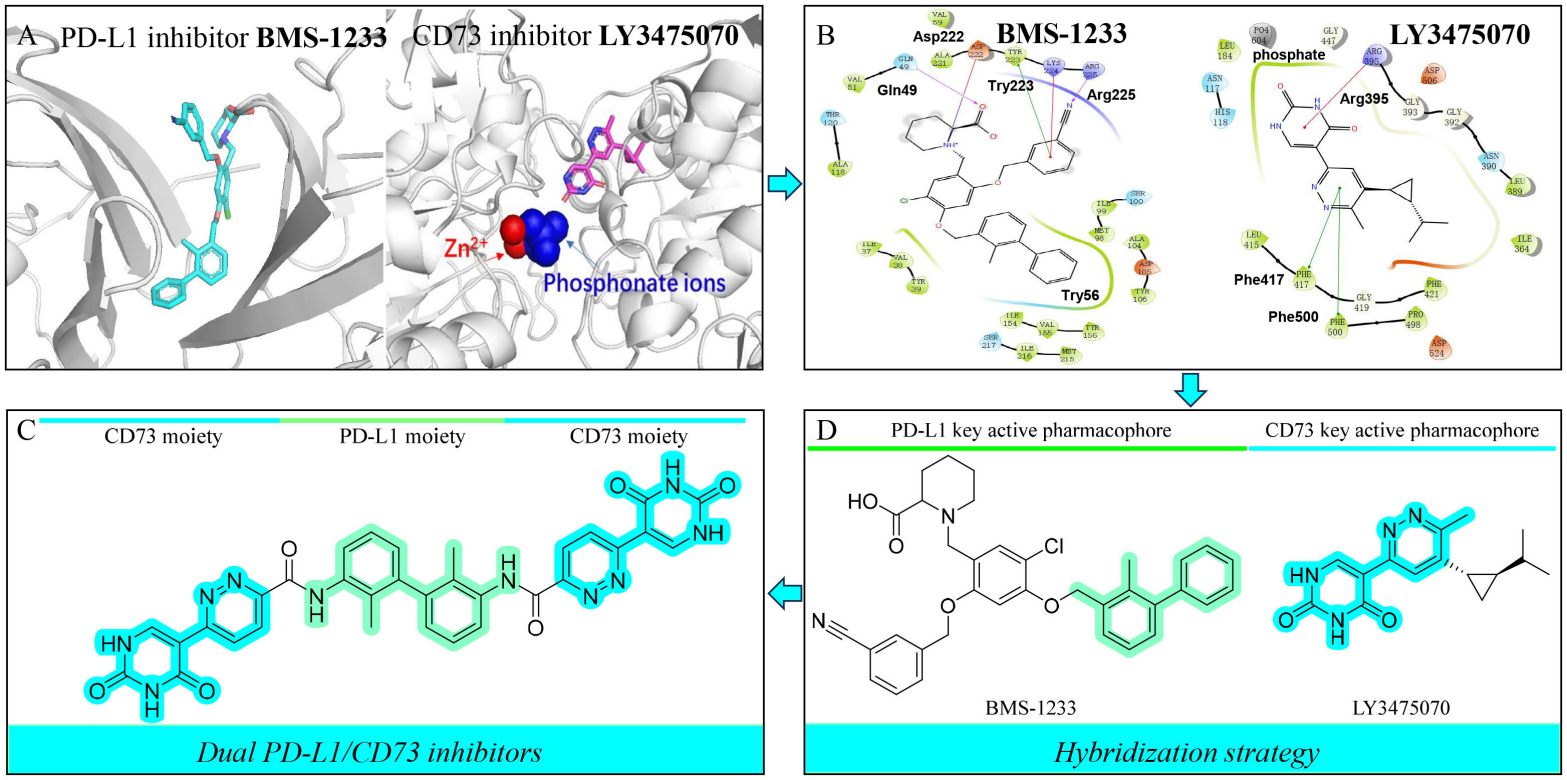
Structure-guided rational design workflow for bifunctional PD-L1/CD73 inhibitors. **(A, B)** Molecular docking binding poses of the reference PD-L1 inhibitor BMS-1233 (PDB: 6RPG) and clinical CD73 inhibitor LY3475070 (PDB: 6XUE) bound to their respective protein pockets. **(C, D)** A hybrid pharmacophore fusion strategy is used to construct dual-target bifunctional scaffolds by linking PD-L1-targeting biphenyl fragments and CD73-binding pyridinedione moieties. The resultant single molecule simultaneously blocks both immunosuppressive signaling cascades in the TME.

To construct bifunctional PD-L1/CD73 inhibitors, we adopted a structure-guided rational design strategy based on high-resolution cocrystal structures of representative inhibitors bound to their respective targets. As illustrated in **Figures 3A, B**, molecular docking simulations validated against published cocrystal structures demonstrate that the prototypical small-molecule PD-L1 inhibitor BMS-1233 inserts its biphenyl scaffold into the hydrophobic cavity at the PD-L1 homodimer interface. This binding mode is stabilized by π–π stacking interactions with Tyr56 and hydrogen bonds formed with Arg225. Meanwhile, the clinical-stage CD73 inhibitor LY3475070 occupies the enzyme’s catalytic pocket, coordinating with active-site Zn²^+^ ions and forming π–π stacking contacts with Phe417 and Phe500 to competitively block AMP hydrolysis into adenosine.

Based on these structurally resolved binding modes, two target-specific pharmacophores were identified: (i) the biphenyl fragment derived from BMS-1233, which mediates high-affinity recognition of the PD-L1 dimer interface; and (ii) the pyridinedione core of LY3475070, which mimics the ribose–phosphate geometry of AMP and chelates catalytic Zn²^+^ ions within the CD73 active site. Using this dual-pharmacophore framework, we designed a series of hybrid molecules through systematic linker optimization. Linker structures were tuned to balance conformational flexibility/rigidity, aqueous solubility, and steric compatibility, enabling covalent fusion of the two pharmacophores without disrupting their native target-binding conformations. The resulting bifunctional compounds simultaneously occupy the immune checkpoint pocket of PD-L1 and suppress the enzymatic activity of CD73. This dual-mode action coordinately blocks two independent immunosuppressive pathways: PD-1/PD-L1 signaling and adenosine accumulation within the TME (**Figures 3C, D**).

### 3.2 Chemical synthesis of CP-1

**Scheme 1:**
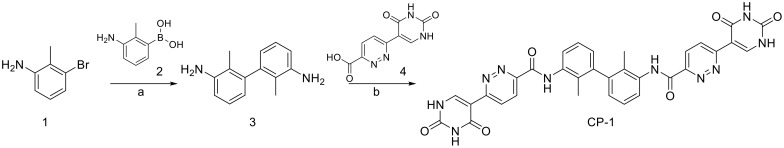
Synthetic route for bifunctional CD73/PD-L1 inhibitor CP-1.

The synthetic route for the CD73/PD−L1 dual inhibitor CP−1 is outlined in **Scheme 1**. First, commercially available 3-bromo-2-methylaniline (intermediate 1) underwent a palladium-catalyzed Suzuki–Miyaura cross-coupling reaction with 3-amino-2-methylphenylboronic acid (intermediate 2) to afford the key biphenyl intermediate 3 (2-methyl-3’-amino-[1,1’-biphenyl]-3-amine). Subsequent amide coupling between intermediate 3 and carboxylic acid-functionalized pyridazinone derivative 4 was performed using HATU as the coupling reagent with DIPEA as the organic base, furnishing the target bifunctional inhibitor CP-1. The chemical structure of CP-1 was unambiguously confirmed via ¹H and ¹³C NMR spectroscopy, and HRMS data matched the theoretical molecular ion mass. HPLC analysis verified that the chemical purity of CP-1 exceeded 95%, satisfying the quality requirements for all subsequent *in vitro* biological assays.

### 3.3 CP-1 exhibits potent dual biochemical inhibitory activity

**Figure 4:**
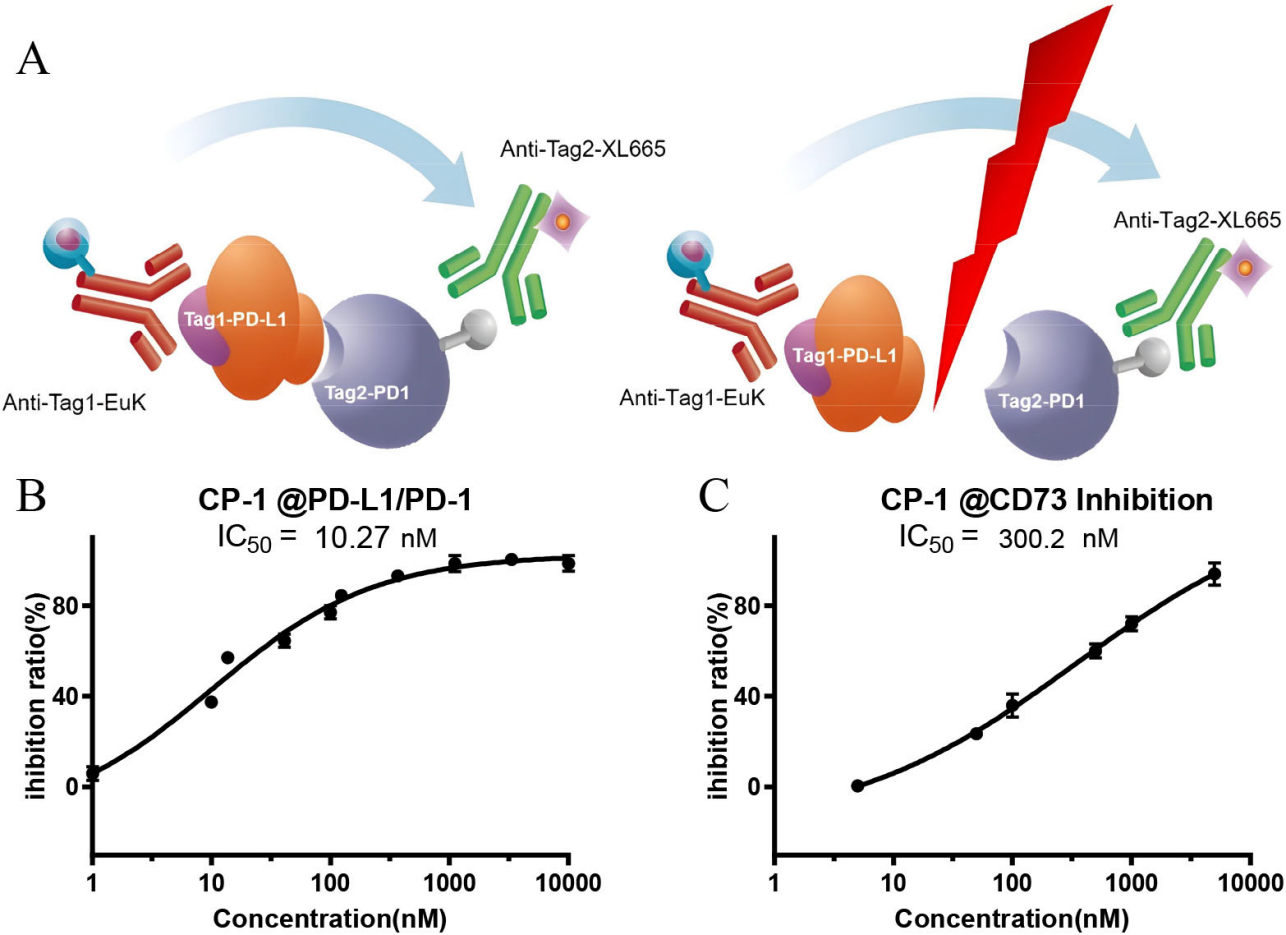
*In vitro* dual biochemical inhibitory activity of lead compound CP-1 measured via HTRF and colorimetric enzymatic assays. **(A)** Schematic principle of the HTRF platform detecting PD-L1 inhibition. **(B)** Concentration-dependent inhibition curve of CP-1 against PD-L1, with a fitted IC_50_ value of 10.27 nM. **(C)** Dose-response curve showing CP-1-mediated suppression of CD73 enzymatic activity, with an IC_50_ of 300.2 nM. CP-1 exhibits balanced nanomolar/submicromolar dual-target potency at the molecular level. The data are derived from three independent experiments. All quantitative data are presented as mean ± standard deviation (SD) unless specified otherwise. Tag1-EuK is the europium cryptate (EuK, HTRF donor fluorophore)-labelled anti-Tag1 antibody, while Tag2-XL665 denotes the XL665 (modified allophycocyanin HTRF acceptor fluorophore)-conjugated anti-Tag2 antibody. Tag1 and Tag2 are unique fusion tags on the two interacting proteins, and the paired fluorophore conjugates enable time-resolved FRET quantification of target protein binding.

To verify the dual-target capacity of our designed scaffold, we first characterized the lead compound CP-1 using well-established biochemical assays against PD-L1 and CD73. PD-L1 blockade was quantified via HTRF assay (**Figures 4A, B**). CP-1 inhibits PD-L1 in a concentration-dependent manner with an IC_50_ of 10.27 ± 4.49 nM, outperforming the positive control BMS-202 (IC_50_ = 61.0 nM). Parallel CD73 enzymatic activity assays also demonstrated dose-dependent inhibitory effects of CP-1, yielding an IC_50_ value of 300.20 ± 13.15 nM. For comparison, the clinical-stage CD73 inhibitor LY-3475070 (currently under Phase I clinical evaluation) exhibited an IC_50_ of 28.1 nM (**Figure 4C**).

Notably, CP-1 achieves balanced dual inhibitory potency, with nanomolar activity against PD-L1 and submicromolar activity against CD73. This dual-target profile lays a solid foundation for downstream functional evaluation, as CP-1 can simultaneously interrupt two nonoverlapping immunosuppressive mechanisms: PD-1/PD-L1-mediated T cell inhibition and CD73-catalyzed adenosine generation in the TME. Such dual blockade is expected to produce superior antitumor effects compared with single-target monotherapies.

### 3.4 SPR confirms direct cross-species binding to PD-L1 and CD73

**Figure 5:**
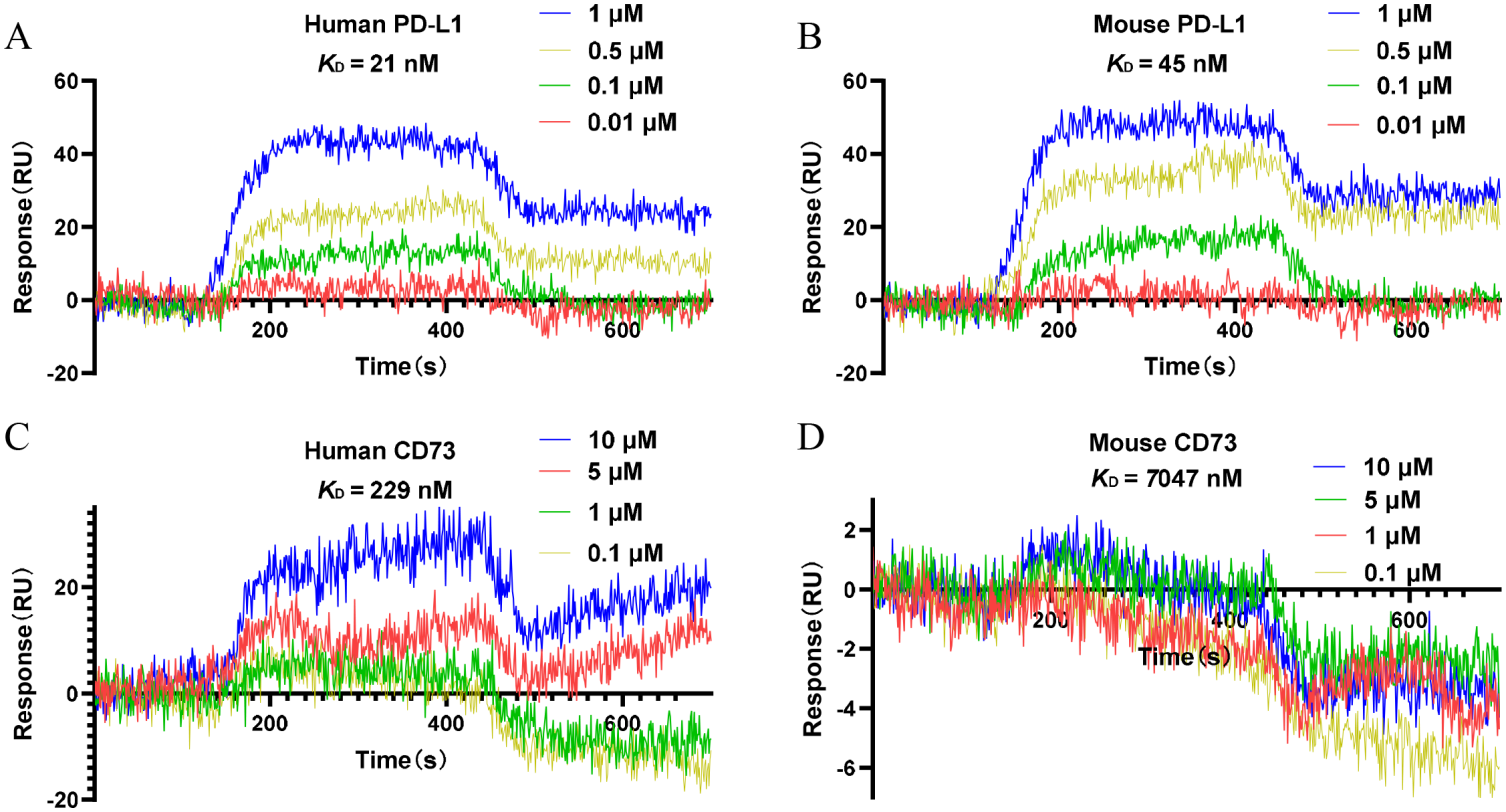
Surface plasmon resonance (SPR) sensorgrams quantifying direct binding affinity of CP-1 to human and murine PD-L1/CD73 orthologs. **(A)** Binding response of CP-1 to human PD-L1 (*K*_D_ = 21 nM); **(B)** Binding profile for murine PD-L1 (*K*_D_ = 45 nM); **(C)** Binding interaction with human CD73 (*K*_D_ = 229 nM); **(D)** Weak cross-species binding to murine CD73 (*K*_D_ = 7047 nM). CP-1 displays robust cross-reactivity toward human and mouse PD-L1, supporting *in vivo* evaluation in immunocompetent mouse models.

Having validated CP-1’s dual biochemical inhibitory activity, we applied surface plasmon resonance (SPR), an orthogonal biophysical technique, to characterize its direct binding to human and murine PD-L1/CD73 orthologs. As shown in **Figures 5A**, CP-1 binds to human PD-L1 (hPD-L1) with high affinity, yielding an equilibrium dissociation constant (*K*_D_) of 21 nM. Similarly, CP-1 exhibited robust binding to murine PD-L1 (mPD-L1; **Figure 5B**), with a *K*_D_ of 45 nM, demonstrating cross-species reactivity toward this immune checkpoint target.

For CD73, SPR measurements revealed that CP-1 binds human soluble CD73 (hCD73; **Figure 5C**) with a *K*_D_ of 229 nM, consistent with its cellular enzymatic IC_50_ value. Binding affinity toward murine CD73 (mCD73; **Figure 5D**) was substantially weaker (*K*_D_ = 7047 nM), a large discrepancy originating from sequence and structural divergence between human and mouse CD73 orthologs, especially within the catalytic and allosteric ligand-binding pockets of the ectoenzyme. This prominent species disparity in CD73 engagement introduces an important interpretive limitation for further mouse *in vivo* studies: CP-1 cannot effectively suppress murine CD73-mediated adenosine production in mice, meaning any antitumor efficacy observed in immunocompetent mouse models will predominantly stem from its robust inhibition of the PD-L1 checkpoint axis, with minimal contribution from CD73 pathway blockade in murine tissues. Consequently, the therapeutic synergy between dual PD-L1/CD73 suppression observed in human *in vitro* systems cannot be fully recapitulated within the mouse experimental setting. This cross-species target-binding gap underscores that our murine efficacy data primarily validate CP-1’s PD-L1 inhibitory function, while definitive preclinical verification of its dual-target synergistic activity will require follow-up studies using humanized CD73/PD-L1 mouse models or human tumor xenograft systems reconstituted with human immune cells. Collectively, these SPR results provide quantitative biophysical evidence that CP-1 engages both human PD-L1 and human CD73 with functionally relevant binding affinity. Its potent cross-reactivity with murine PD-L1 alone supports the partial pharmacological suitability of CP-1 for preliminary *in vivo* efficacy testing in immunocompetent mouse models, yet its weak mCD73 binding limits a full assessment of its dual synergistic mechanism in wild-type mice without humanized target expression.

### 3.5 CP-1 restores T cell function in PD-1/PD-L1 NFAT reporter assay

**Figure 6:**
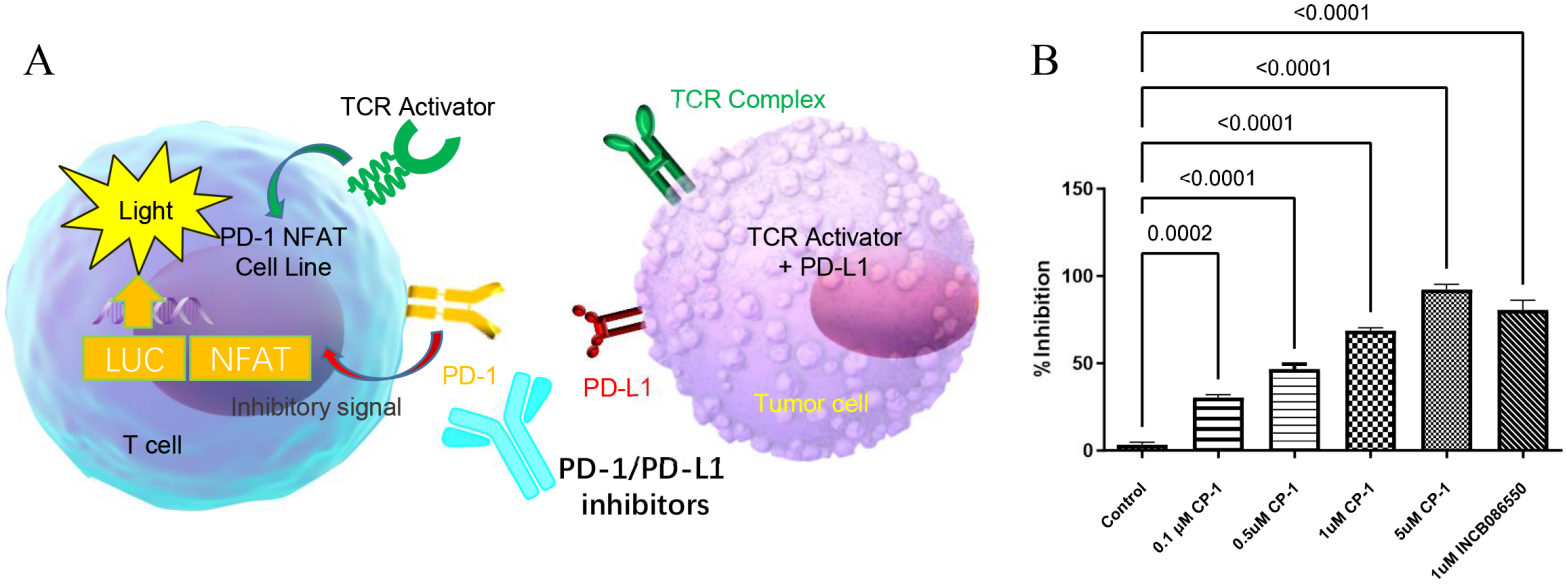
Cellular functional validation of CP-1 in the PD-1/PD-L1 NFAT reporter coculture assay. **(A)** Schematic workflow of the dual-cell reporter system that recapitulates PD-L1-dependent T cell suppression. **(B)** Quantitative comparison of T cell rescue activity between benchmark PD-L1 inhibitor INCB086550 and CP-1 across gradient concentrations. CP-1 restores T cell transcriptional activity with an EC_50_ of 0.9 μM, confirming intact PD-L1 blockade function in cells. LUC: luciferase; NFAT: nuclear factor of activated T cells; TCR: T cell receptor. The data are derived from three independent experiments. All quantitative data are presented as mean ± standard deviation (SD) unless specified otherwise. Statistical comparisons were performed using one-way analysis of variance (ANOVA). Significance thresholds were defined as *P < 0.05, **P < 0.01, ***P < 0.001, ****P < 0.0001.

We next assessed the cellular immunomodulatory function of CP-1 using a well-established PD-1/PD-L1 NFAT reporter coculture system, which recapitulates PD-L1-mediated T cell suppression (**Figure 6A**). In this coculture model, PD-L1-expressing tumor cells inhibit TCR-triggered NFAT nuclear translocation and downstream luciferase reporter expression in PD-1-overexpressing Jurkat T cells. Effective PD-1/PD-L1 blockade reverses this inhibitory phenotype and restores NFAT-driven luminescence, serving as a quantitative readout of recovered T-cell effector function (34).

As presented in **Figures 6B**, CP-1 induced a clear concentration-dependent elevation in NFAT reporter activity, confirming its capacity to disrupt PD-1/PD-L1-mediated T cell suppression at the cellular level. Significant functional recovery was observed even at submicromolar concentrations (*p* < 0.0001 vs. vehicle control). At 1 μM, CP-1 exhibited comparable cellular activity to the clinical-stage small-molecule PD-L1 inhibitor INCB086550, a benchmark immunotherapeutic agent. The calculated cellular EC_50_ of CP-1 in this reporter assay was 0.9 μM, verifying functional PD-1/PD-L1 blockade at pharmacologically achievable concentrations.

Combined with the preceding biochemical and SPR data, these cellular results highlight CP-1’s strong translational potential. Unlike many dual inhibitors that display potent target binding in biochemical assays yet fail to translate into cellular activity, CP-1 retains robust PD-L1 inhibitory function within complex cellular environments, supporting its further development as a lead bifunctional immunotherapeutic agent.

### 3.6 CP-1 boosts T cell antitumor function and blocks CD73-dependent adenosine production *in ex vivo* models

**Figure 7:**
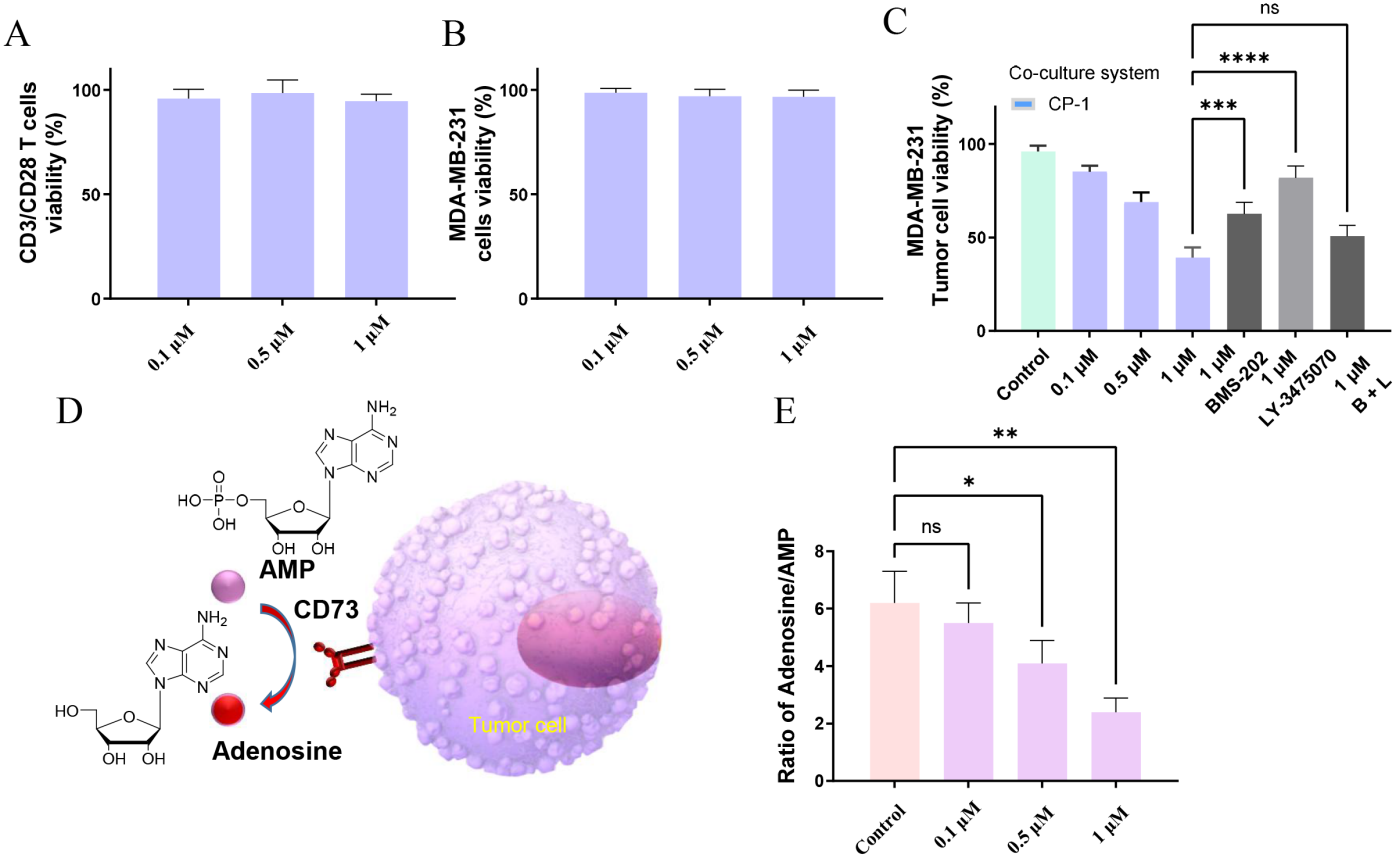
CP-1 restores T cell effector function and induces dose-dependent tumor cell killing *in ex vivo* MDA-MB-231–CD3/CD28 T cell co-cultures. CCK-8 cell viability assays were performed to evaluate the cytotoxicity of CP-1 against isolated cell populations and its immunotherapeutic activity in co-culture models. **(A)** Viability of purified CD3/CD28-activated T cells treated with 0.1–1 μM CP-1; no significant reduction in T cell survival was observed across all tested concentrations. **(B)** Viability of monocultured MDA-MB-231 tumor cells incubated with 0.1–1 μM CP-1, demonstrating that CP-1 lacks direct intrinsic cytotoxicity toward breast tumor cells alone. **(C)** Quantified MDA-MB-231 tumor cell viability within the T cell-tumor co-culture system. CP-1 inhibited Adenosine production. **(D)** Mechanism of action of CD73 mediating the conversion of AMP to Adenosine; **(E)** significant Adenosine modulation. Compared with the untreated control group, CP-1 treatment induced a gradual, concentration-dependent decline in tumor cell viability. The data are derived from three independent experiments. Statistical comparisons were performed using one-way analysis of variance (ANOVA). Significance thresholds were defined as *P < 0.05, **P < 0.01, ***P < 0.001, ****P < 0.0001.

To further verify whether bifunctional CP-1 triggers robust functional immune activation by simultaneously suppressing PD-L1 and CD73, we evaluated T cell-dependent tumour-killing effects using an *ex vivo* MDA-MB-231 tumour–CD3/CD28 T cell co-culture system. Isolated CD3/CD28-activated T lymphocytes were co-incubated with MDA-MB-231 breast cancer cells in the presence of serially diluted CP-1 (0.1, 0.5, and 1 μM). First, single-cell monoculture cytotoxicity tests were performed to exclude direct cell-intrinsic toxicity of CP-1. As shown in **Figures 7A, B**, treatment with 0.1–1 μM CP-1 did not reduce the viability of purified CD3/CD28 T cells or of monocultured MDA-MB-231 tumour cells, confirming that CP-1 lacks direct killing effects on either cell population.

In the tumour-T cell co-culture setting (**Figure 7C**), vehicle-treated control groups showed robust tumour cell survival, whereas CP-1 treatment induced a gradual, concentration-dependent reduction in MDA-MB-231 viability. We included parallel reference groups comprising single-target inhibitors (1 μM BMS-202, a selective PD-L1 inhibitor; 1 μM LY-3475070, a clinical-stage CD73 inhibitor) and their combination (B + L) to dissect the individual and combined contributions of PD-L1 and CD73 blockade. Both single agents achieved only mild suppression of tumour growth. At 1 μM, CP-1 reduced tumour cell viability to 39.2%, which was moderately superior to the combination group (50.6% tumour cell viability), indicating modestly enhanced T cell-mediated tumoricidal activity from dual PD-L1/CD73 engagement within a single molecule.

Beyond PD-L1 checkpoint interference, we next characterised CP-1’s functional inhibition of CD73 enzymatic activity in tumour cells. Membrane-tethered CD73 acts as a rate-limiting ectoenzyme that catalyses the hydrolysis of extracellular AMP to immunosuppressive adenosine; pharmacological CD73 blockade interrupts this metabolic cascade, thereby reducing intratumoural adenosine levels. To confirm CP-1-mediated CD73 suppression at the cellular level, we quantified the intracellular adenosine/AMP ratio in MDA-MB-231 cells. **Figure 7D** schematically illustrates the canonical CD73-catalysed AMP-to-adenosine conversion pathway. Consistent with our biochemical CD73 activity assay results, CP-1 treatment dose-dependently decreased the adenosine/AMP ratio relative to the vehicle control group (**Figure 7E**), validating that CP-1 effectively impedes CD73-dependent adenosine generation in intact tumour cells.

Taken together, these *ex vivo* co-culture and cellular metabolite measurements demonstrate that CP-1 elicits stronger T cell-dependent anti-tumour activity through concurrent inhibition of both the PD-L1 immune checkpoint and the CD73-adenosine immunosuppressive axis. Its unified dual-target design yields moderately improved immunotherapeutic potency compared with separate co-administration of two single-target small-molecule inhibitors, supporting the functional advantage of dual PD-L1/CD73 co-inhibition.

### 3.7 Molecular docking analysis of CP−1 with PD−L1 and CD73

**Figure 8:**
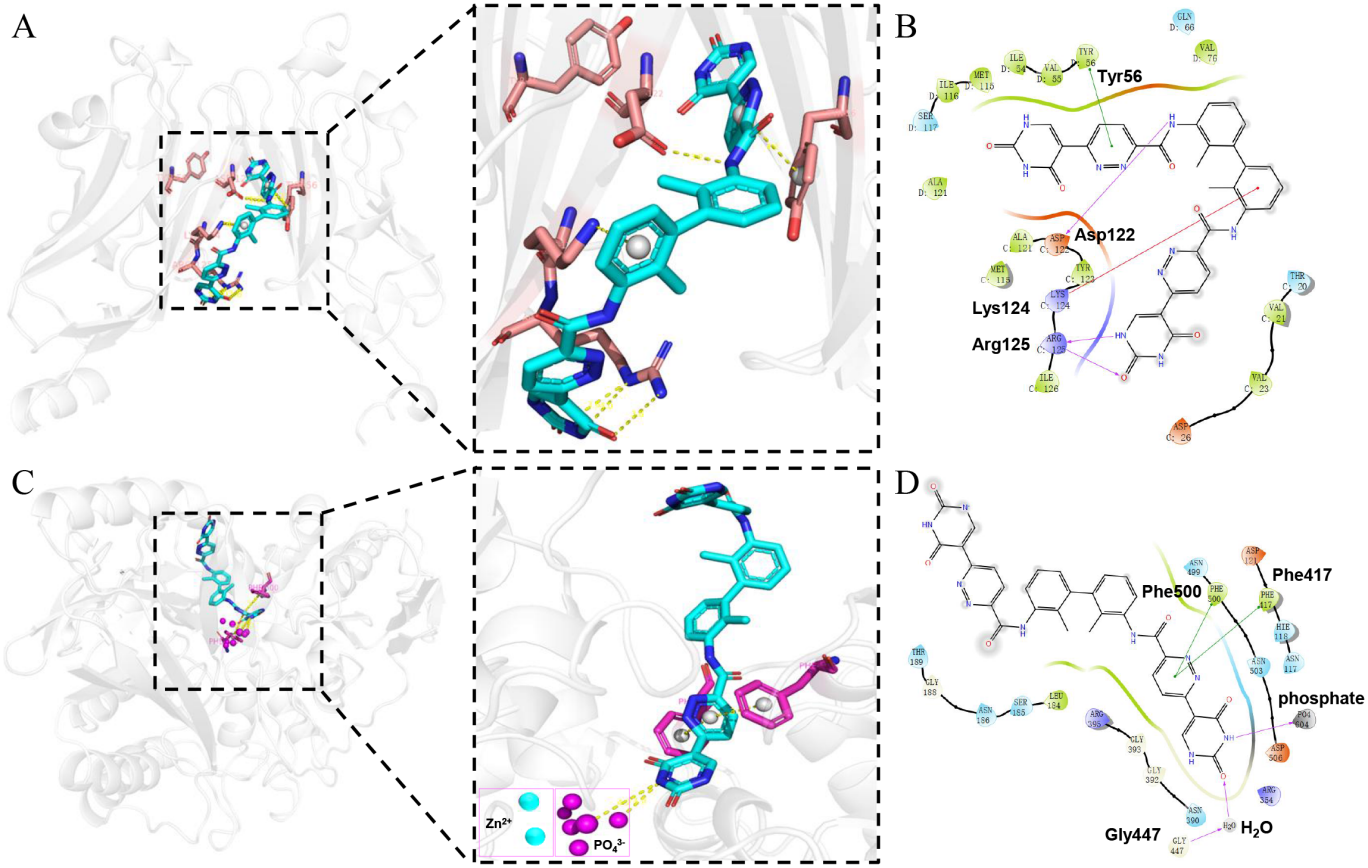
Molecular docking analysis revealing the dual binding modes of CP-1 with human PD-L1 and CD73. **(A, B)** CP-1 inserts its biphenyl core into the hydrophobic homodimer cavity of PD-L1 and forms stabilizing hydrogen bonds with pocket residues. **(C, D)** The pyridinedione pharmacophore chelates catalytic Zn²^+^ ions within the CD73 active site to competitively block AMP substrate binding. Distinct dual-pharmacophore interactions account for the balanced dual-target inhibitory potency of CP-1. Zn²^+^ ions are marked by solid blue-filled circles.

To rationalize the potent dual−target activity of CP−1 observed in biochemical and cellular assays, we performed molecular docking simulations against human PD−L1 (PDB: 6RPG) and human CD73 (PDB: 6XUE). When bound to PD-L1, CP-1 adopts a binding conformation analogous to the reference inhibitor BMS-1233, with its central biphenyl scaffold inserted into the hydrophobic cavity at the PD-L1 homodimer interface (**Figures 8A, B**). The biphenyl core forms extensive π−π stacking interactions with key aromatic residues, while the linker and terminal moieties form additional hydrogen bonds to stabilize the protein-ligand complex. Notably, the pyrimidine−2,4−dione fragment, originally designed as the CD73 pharmacophore, forms a hydrogen bond with Arg125 of PD−L1, contributing to a favorable binding conformation and high predicted docking score. This bifunctional scaffold design enables CP−1 to occupy the canonical PD−L1 binding pocket while forming extra stabilizing interactions, accounting for its potent inhibition of the PD−1/PD−L1 axis. For CD73, CP−1 adopts a binding pose similar to the clinical inhibitor LY3475070, effectively occupying the enzyme’s catalytic pocket (**Figures 8C, D**). The pyrimidine−2,4−dione core forms a stable “sandwich” coordination complex with the catalytic Zn²^+^ ions via phosphate−mediated hydrogen bonds, directly competing with AMP for active site occupancy. This interaction blocks the conversion of AMP into immunosuppressive adenosine, consistent with the measured enzymatic inhibitory activity. The biphenyl PD−L1−binding fragment extends outward into the solvent−exposed surface, forming additional weak intermolecular contacts that further anchor the ligand within the pocket. These docking simulations provide a structural mechanistic basis for CP-1’s balanced dual−target activity. CP-1 interacts with both PD-L1 and CD73 through specific, well-defined noncovalent contacts that align with its *in vitro* potency profiles. The computational results validate our rational molecular hybridization strategy and explain how a single bifunctional scaffold can simultaneously disrupt two core immunosuppressive pathways within the TME.

## 4 Discussion

First-generation immune checkpoint inhibitors, primarily monoclonal antibodies targeting the PD-1/PD-L1 axis, have revolutionized the landscape of cancer treatment. However, their clinical utility is severely limited by low objective response rates (typically below 20%), intrinsic and acquired therapeutic resistance, and poor activation of antitumor immunity in immunologically “cold” tumors. Although combinatorial regimens pairing PD-L1 inhibitors with chemotherapy, targeted therapies, or other immunomodulators have generated promising synergistic effects in clinical trials, multi-drug combination strategies carry unavoidable drawbacks: overlapping systemic toxicities, mismatched pharmacokinetic profiles, inconsistent intratumoral drug distribution, and unpredictable drug–drug interactions, all of which hinder clinical translation. Against this backdrop, rationally designed small-molecule dual-target inhibitors that simultaneously block PD-L1 and a functionally complementary immunosuppressive pathway have emerged as a promising next-generation immunotherapeutic strategy.

Among all candidate co-targets, CD73 represents an exceptionally attractive partner due to its extensive mechanistic crosstalk with the PD-1/PD-L1 axis. CD73 drives the conversion of extracellular AMP to adenosine, which signals through adenosine receptors on immune cells to suppress T cell activation, amplify Treg suppressive function, and inhibit NK cell cytotoxicity. This creates a profoundly immunosuppressive microenvironment that acts in parallel with PD-L1-mediated immune escape. Critical preclinical and clinical evidence confirms that CD73 inhibition not only reduces adenosine-driven immunosuppression but also potentiates the efficacy of PD-L1 blockade, as dual co-targeting disrupts two nonredundant immunosuppressive cascades. Conversely, PD-L1 monotherapy often fails to fully reverse T cell exhaustion in the presence of high extracellular adenosine concentrations, which helps explain the limited response rates observed with single-agent immunotherapy. This bidirectional functional synergy provides a robust mechanistic rationale for co-targeting CD73 and PD-L1.

Numerous prior studies have described bifunctional small molecules built around the PD-L1 scaffold, including PD-L1/HDAC, PD-L1/PARP1, and PD-L1/IDO1 inhibitors, all of which employ a dual-pharmacophore hybridization strategy to elicit synergistic antitumor immune responses. Our group previously reported CC-5 as a dual PD-L1/CD73 bifunctional inhibitor CC-5. CC-5 exhibited robust PD-L1 binding activity with an IC_50_ of 6 nM and moderate CD73 enzymatic inhibitory potency at an IC_50_ of 0.773 μM. In the present work, we identified CP-1 as a novel dual-target inhibitor with markedly improved balanced dual inhibitory profiles. CP-1 disrupts PD-1/PD-L1 interaction with an IC_50_ of 10.27 nM and suppresses CD73 catalytic activity at an IC_50_ of 300.2 nM, representing a nearly 2-fold enhancement in CD73 inhibitory potency relative to CC-5.

Furthermore, CP-1 displays superior druglike properties compared with CC-5. CC-5 possesses a molecular weight of 837.381, a Crippen LogP value of 5.918, 10 hydrogen bond acceptors, 2 hydrogen bond donors, and a TPSA of 160.54. In contrast, CP-1 features a reduced molecular weight of 644.608, a drastically lowered Crippen LogP of 0.215, 10 hydrogen bond acceptors, 6 hydrogen bond donors, and an elevated TPSA of 241.2. The optimized physicochemical parameters of CP-1 effectively reduce lipophilicity and molecular size, thereby greatly improving its overall drug-likeness and mitigating the suboptimal pharmacokinetic risks associated with the highly lipophilic, high-molecular-weight CC-5 scaffold. Most existing PD-L1 bifunctional inhibitors combine the checkpoint binder with epigenetic or DNA repair regulators, whereas CP-1 simultaneously interferes with two independent immune-suppressive cascades: the PD-1/PD-L1 checkpoint and CD73-mediated adenosine metabolism, thereby potentially triggering more direct and coordinated immune activation. Bispecific antibodies co-targeting CD73 and PD-L1 have also been shown to elicit potent antitumor immunity, yet antibody-based modalities are plagued by inherent drawbacks, including immunogenicity, poor penetration into dense solid tumor tissue, and complex manufacturing workflows. By comparison, small-molecule CP-1 possesses low molecular weight and scalable synthetic routes, conferring superior translational prospects relative to antibody therapeutics. When benchmarked against single-selective inhibitors, including BMS-1233 for PD-L1 and LY-3475070 for CD73, CP-1 circumvents the inconsistent pharmacokinetics and additive toxic risks associated with co-administration of separate drugs, delivering dual pathway suppression via a single chemical entity. It is also worth noting that CP-1 exhibits weaker CD73 enzymatic inhibition than dedicated single-agent CD73 inhibitors, an observation that can be rationalized by structural trade-offs introduced during molecular hybridization. The rigid amide linker used to accommodate PD-L1 pocket binding distorts the optimal binding conformation of the pyridinedione core within the CD73 catalytic cleft, weakening its chelating interactions with active-site zinc ions; this structural compromise to balance dual-target affinity also provides clear clues for subsequent scaffold optimization.

Compared with antibody therapeutics and multi-drug combination regimens, small-molecule CD73/PD-L1 dual inhibitors possess unique translational advantages. As single chemical entities, they exhibit unified, predictable pharmacokinetic behavior, synchronized intratumoral accumulation, and enhanced tissue penetration—properties that are particularly valuable for poorly vascularized solid tumors, where antibodies cannot reach therapeutic concentrations. Furthermore, bifunctional small molecules eliminate the risk of overlapping toxicities and complex drug–drug interactions associated with combination therapy. Collectively, the development of bifunctional CD73/PD-L1 small-molecule inhibitors represents a rational and powerful approach to overcoming immunotherapy resistance, broadening the therapeutic index, and expanding the pool of patients eligible for effective cancer immunotherapy.

Despite the favorable dual-target potency and cellular immunomodulatory activity observed for CP-1, several notable limitations of the present study should be acknowledged. All biological characterizations in this work are confined to *in vitro* biochemical, biophysical, and cellular assays, and we have not yet evaluated the *in vivo* antitumor efficacy or systemic toxicological safety of CP-1 in animal models. This investigation provides a comprehensive profiling of a single lead molecule, CP-1, but lacks systematic structure–activity relationship analysis of linker length, rigidity, and aromatic substituents to guide further potency improvement. We also did not quantify changes in extracellular adenosine levels or in downstream A2a receptor-mediated immune signaling after compound treatment, which limits deeper mechanistic validation of CP-1’s intracellular CD73-inhibitory effects. Next, we will design an analog series equipped with flexible linkers of variable lengths to relieve steric conflicts, strengthening the pyridinedione fragment’s coordination with CD73’s zinc active site without sacrificing high PD-L1 affinity. A full library of modified derivatives with diverse linkers and terminal aromatic groups will be synthesized to establish complete structure–activity relationships, enabling the identification of second-generation leads with balanced nanomolar potency against both targets. Beyond single-agent evaluation, we will further explore combinatorial regimens pairing optimized bifunctional inhibitors with radiotherapy or chemotherapy to maximize synergistic antitumor immunotherapeutic benefits.

## 5 Conclusions

In this study, we applied structure-based rational drug design to develop CP-1, an innovative bifunctional small molecule that simultaneously inhibits PD-L1 and CD73. Biochemical assays confirmed balanced dual-target inhibitory activity: CP-1 potently blocks PD-1/PD-L1 protein–protein interaction with an IC_50_ of 10.27 nM and markedly suppresses CD73 enzymatic activity with an IC_50_ of 300.2 nM. Biophysical SPR binding experiments further confirmed that CP-1 directly interacts with human and murine PD-L1/CD73, demonstrating reliable cross-species binding. Cell-based NFAT reporter functional assays confirmed that CP-1 effectively restores effector function in T cells suppressed by PD-1/PD-L1 checkpoint blockade, thereby exerting prominent immunomodulatory activity at the cellular level. Molecular docking simulations elucidated the dual binding modes, revealing that CP-1 forms stable noncovalent interactions with key amino acid residues in the PD-L1 homodimer cavity and in the catalytic domain of CD73. Its two independent pharmacophores anchor to the respective active sites via hydrogen bonds and metal coordination contacts, fully accounting for its strong dual-target binding affinity and inhibitory potency.

Taken together, these results establish CP-1 as a viable lead scaffold for dual PD-L1/CD73 cancer immunotherapy. This bifunctional agent simultaneously alleviates two major forms of immunosuppression within the tumor microenvironment: reversing PD-L1-induced T cell exhaustion and attenuating CD73-mediated adenosine-dependent immune tolerance, thereby providing an effective solution to the therapeutic bottlenecks of single-target immunotherapy.

## Data Availability

The datasets presented in this study can be found in online repositories. The names of the repository/repositories and accession number(s) can be found in the article/**Supplementary Material**.
