# Supplementary material for: Dual-targeting CD73/PD-L1 bifunctional inhibitor: a promising cancer immunotherapy strategy
Source: Front Immunol. 2026 Jul 23;17:1898645. doi: 10.3389/fimmu.2026.1898645 (PMC13442810; doi:10.3389/fimmu.2026.1898645)
Supplement: Supplementary file 1 [file DataSheet1.pdf]

Supporting Information

Dual-Targeting CD73/PD-L1 Bifunctional Inhibitor: A Promising Cancer Immunotherapy Strategy

Jingjing Du[1,#], Sen Wu[2,#], Shiyun Cheng[1], Xiaobo Zeng[1,*], and Binbin Cheng[1, *]

[1]Hubei Provincial Key Laboratory of Occurrence and Intervention of Kidney Diseases, Hubei Provincial Engineering Research Center of Immunotherapy Drugs for Renal Tumors, Hubei Polytechnic University, School of Medicine, Huangshi, Hubei, 435003, China.

[2]Hubei Institute for Drug Control, Hubei Institute of Biological Products, Wuhan, Hubei, 430075, China.

# These authors contributed equally to this article.

* Correspondence: Xiaobo Zeng: xbzeng2013@126.com; Binbin Cheng: 2248549496@qq.com.

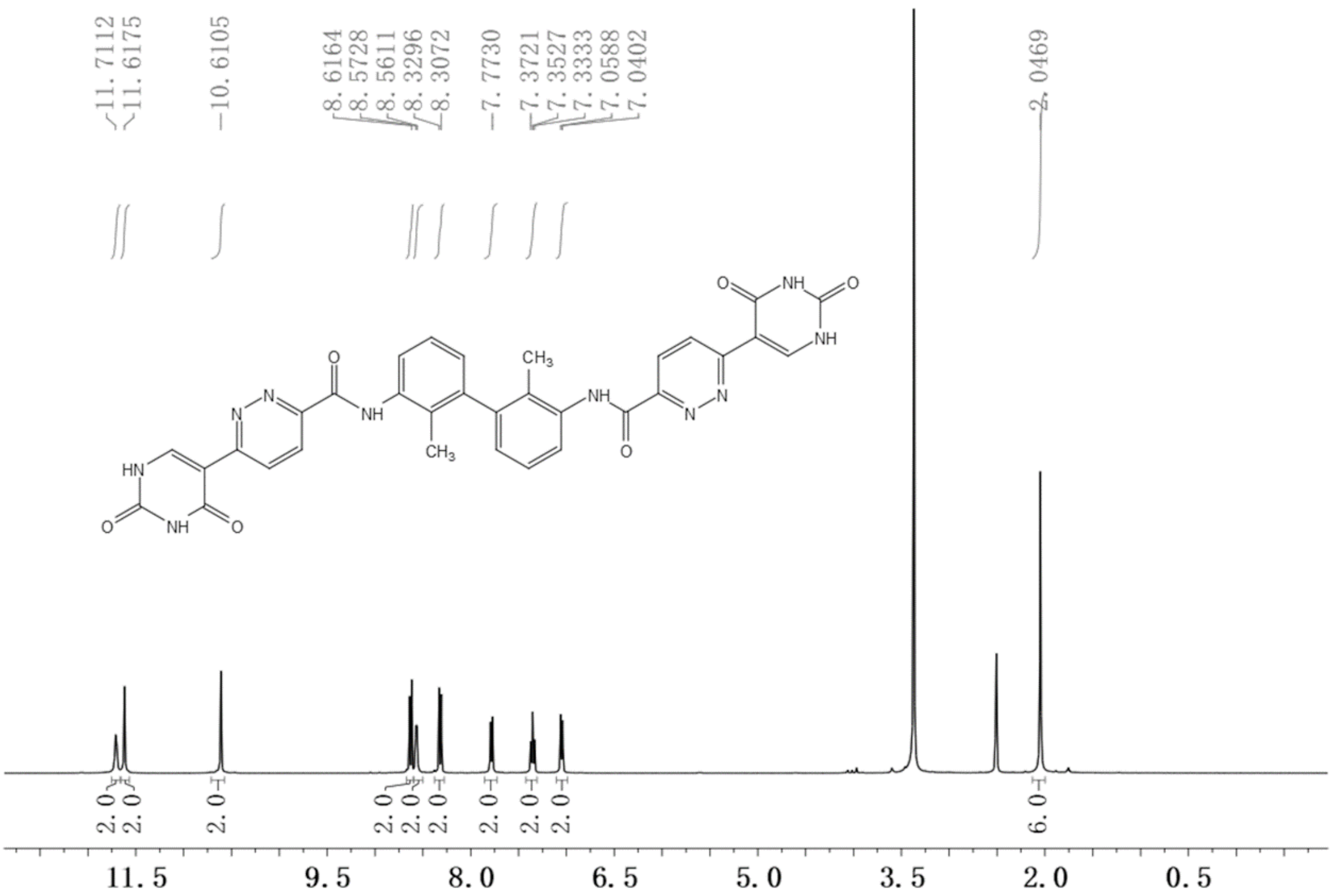
11.7112
11.6175
10.6105
8.6164
8.5728
8.5611
8.3296
8.3072
7.7730
7.3721
7.3527
7.3333
7.0588
7.0402
2.0469
CH3
NH
O
N
HN
2.0
2.0
2.0
2.0
2.0
2.0
2.0
2.0
2.0
6.0
11.5
9.5
8.0
6.5
5.0
3.5
2.0
0.5

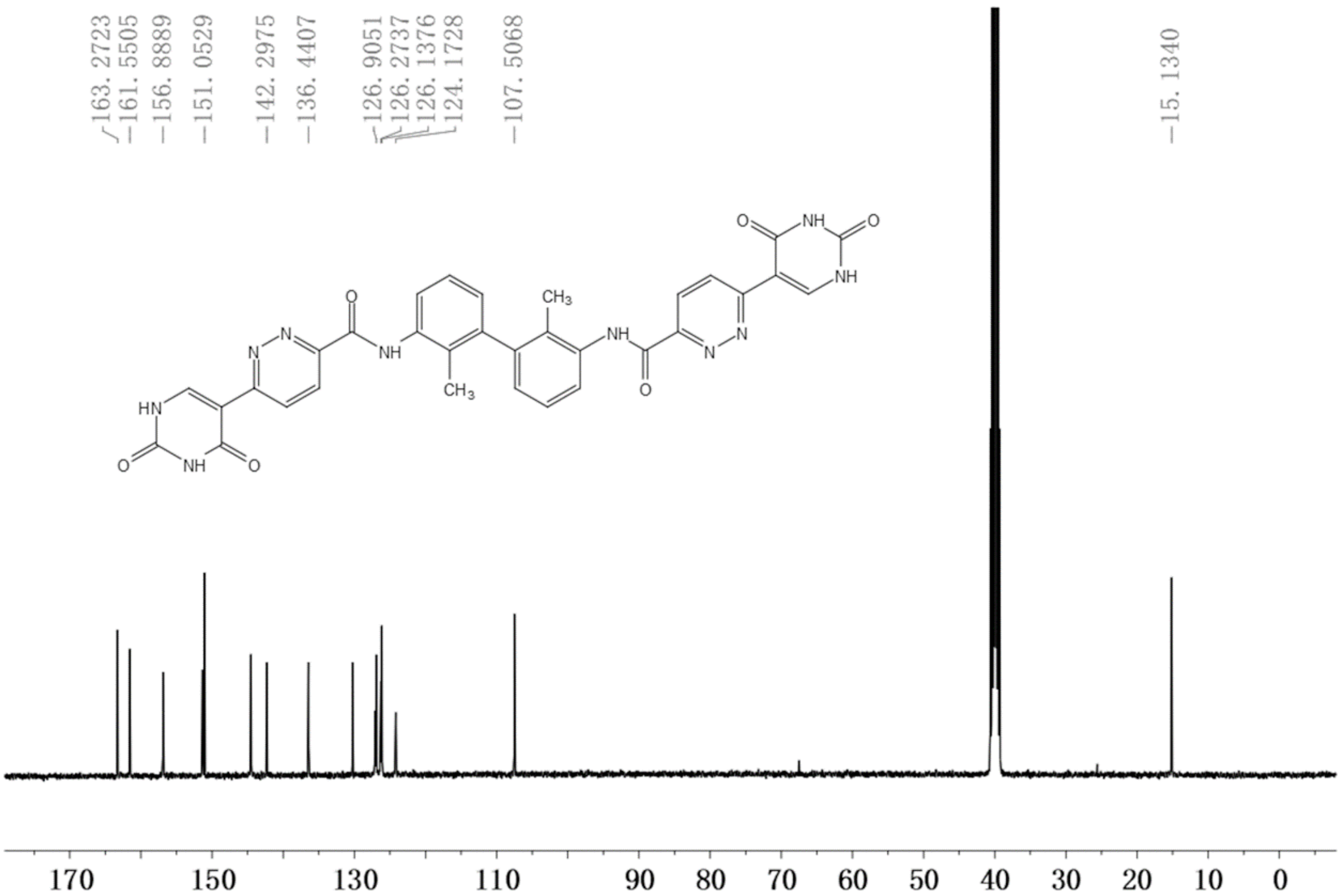
163.2723
161.5505
156.8889
151.0529
142.2975
136.4407
126.9051
126.2737
126.1376
124.1728
107.5068
15.1340
CH3
NH
O
N
HN
170
150
130
110
90
80
70
60
50
40
30
20
10
0
